# T-Cells and Interferon Gamma Are Necessary for Survival Following Crimean-Congo Hemorrhagic Fever Virus Infection in Mice

**DOI:** 10.3390/microorganisms9020279

**Published:** 2021-01-29

**Authors:** David W. Hawman, Kimberly Meade-White, Shanna Leventhal, Aaron Carmody, Elaine Haddock, Kim Hasenkrug, Heinz Feldmann

**Affiliations:** 1 Laboratory of Virology, NIAID/NIH, Hamilton, MT 59840, USA; kmeade-white@niaid.nih.gov (K.M.-W.); shanna.leventhal@nih.gov (S.L.); elaine.haddock@nih.gov (E.H.); 2 Research Technologies Branch, NIAID/NIH, Hamilton, MT 59840, USA; acarmody@niaid.nih.gov; 3 Laboratory of Persistent Viral Diseases, NIAID/NIH, Hamilton, MT 59840, USA; KHASENKRUG@niaid.nih.gov

**Keywords:** Crimean-Congo hemorrhagic fever, CCHFV, T-cells, mouse model, IFNγ

## Abstract

Crimean-Congo hemorrhagic fever (CCHF) is a severe tick-borne febrile illness with wide geographic distribution. In humans, the disease follows infection by the Crimean-Congo hemorrhagic fever virus (CCHFV) and begins as flu-like symptoms that can rapidly progress to hemorrhaging and death. Case fatality rates can be as high as 30%. An important gap in our understanding of CCHF are the host immune responses necessary to control the infection. A better understanding of these responses is needed to direct therapeutic strategies to limit the often-severe morbidity and mortality seen in humans. In this report, we have utilized a mouse model in which mice develop severe disease but ultimately recover. T-cells were robustly activated, differentiated to produce antiviral cytokines, and were critical for survival following CCHFV infection. We further identified a key role for interferon gamma (IFNγ) in survival following CCHFV infection. These results significantly improve our understanding of the host adaptive immune response to severe CCHFV infection.

## 1. Introduction

Crimean-Congo hemorrhagic fever virus (CCHFV) is a tick-borne virus that can cause severe, hemorrhagic disease in humans. Along with its tick vector, ticks of the *Hyalomma* genus, it is widely distributed across Eastern Europe, Africa, the Middle East, and Asia and the geographic range continues to increase [1]. Humans typically become infected following bites by infected ticks, handling of infected livestock, or in the healthcare setting [2]. In humans, Crimean-Congo hemorrhagic fever (CCHF) begins as a non-specific febrile illness that can rapidly progress to a serious hemorrhagic disease. This phase of disease is characterized by thrombocytopenia, high viral loads, inflammatory cytokine production, along with bleeding from various sites around the body [2]. Low platelet count, elevated liver enzymes, absent antibody responses, and elevated levels of inflammatory cytokines such as IL-6 all correlate with poor outcome [3,4,5,6,7]. A significant gap in our understanding of CCHFV pathogenesis is the host responses necessary for control of the infection. Importantly, the role of adaptive immunity in control of CCHFV is unclear. Furthermore, for several other hemorrhagic fevers, dysregulated inflammatory immune responses can contribute substantially to morbidity and mortality [8,9]. Whether similar processes occur in CCHFV-infected humans is not well understood but fatal outcome is often associated with high levels of inflammatory cytokines [6,7], suggesting excessive inflammatory immune responses may contribute to poor outcome.

Our group recently described a mouse model in which type I IFN deficient mice infected with a clinical isolate of CCHFV exhibit severe disease but ultimately recover from the infection [10]. Recovery correlated with development of early and long lasting CCHFV-specific B- and T-cell responses [10]. In this study, we utilized this model to more thoroughly investigate the T-cell response to CCHFV infection. We found that following CCHFV-infection, T-cells are robustly activated, proliferate and differentiate to produce T-helper-1 (Th1)-type cytokines. Furthermore, we found that T-cells are necessary for mice to survive acute CCHFV-infection. Lastly, we identified IFNγ as a key antiviral cytokine in survival following CCHFV-infection.

## 2. Materials and Methods

Biosafety and Ethics. All procedures with infectious CCHFV were conducted at biosafety level 4 (BSL4) conditions in accordance with operating procedures approved by the Rocky Mountain Laboratories institutional biosafety committee. Animal experiments were approved by the institutional animal care and use committee and performed by experienced personnel under veterinary oversight. Mice were group-housed in HEPA-filtered cage systems and acclimatized to BSL4 conditions prior to start of the experiment. They were provided with nesting material and food and water ad libitum. Mice were humanely euthanized according to the following criteria: weight loss >25%, ataxia, extreme lethargy (animal is unresponsive to touch), bloody discharge from nose, mouth, rectum or urogenital area, tachypnea, dyspnea, or paralysis of the limbs. Although we comprehensively evaluated the mice for any of these clinical signs, mice were typically euthanized for weight loss or extreme lethargy. For survival analyses, mice that were euthanized for humane reasons were recorded as succumbing the day following euthanasia.

Mice. IFNAR^−/−^ mice on the C57BL/6 background were from an in-house breeding colony. Mixed-sex 8–12-week-old mice were used for all studies. All mice were inoculated with 1 median tissue-culture infectious dose (TCID_50_), equivalent to 500 median infectious dose for IFNAR^−/−^ mice [10], via a 100 μL subcutaneous injection to the subscapular region. Virus was diluted in sterile DMEM culture media without additives. Mock-infected mice received an equivalent injection of DMEM alone.

In vivo depletions. Mice were treated with 200 µg of rat IgG2b isotype (clone 1-2), α-CD4 (clone GK1.5), α-CD8 (clone Ly2.2), or both diluted in sterile phosphate buffered saline (PBS) via intraperitoneal (IP) injections on day −2, +3, +10, and +17 relative to CCHFV-challenge. All antibodies were purchased from Leinco. In a subset of mice euthanized at day +8 PI, depletion efficacy was monitored by flow cytometry. For B-cell depletion, mice were treated with 200 μg α-CD20, generously provided by Genentech, or mouse IgG2a isotype control (BioXCell) on days −2, +3, +10, +17 relative to CCHFV-challenge. In a subset of mice, on day +5, B-cell depletion was evaluated by flow cytometry.

In vivo IFNγ neutralization and treatment. On day −1, mice were treated with 1 mg of rat IgG1 isotype (α-horseradish peroxidase, clone HRPN, BioXcell) or α-IFNγ (clone XMG1.2, Leinco) diluted in PBS via IP injections. Thereafter, to maintain suppression, mice were treated with 200 µg antibody every 48 h until study end.

Blood chemistry and cytokine assay. Blood was collected at time of euthanasia by cardiac puncture into lithium heparin treated tubes (BD). Liver enzymes were measured on Vetscan 2 analyzers with Preventive Care Profile disks (Abaxis, Union City, CA, USA). Plasma was separated from lithium heparin treated blood by centrifugation and plasma irradiated according to approved procedures to inactivate CCHFV. Cytokines in plasma were then quantified by mouse 23-plex cytokine assay (Biorad, Hercules, CA, USA) according to assay instructions.

Single cell suspensions from liver and spleen. Single cell suspensions from mouse spleens were generated by passaging the spleen through a 70-micron strainer (Sigma, St. Louis, MO, USA) and collected into RPMI media (ThermoFisher, Waltham, MA, USA) supplemented with 10% FBS (ThermoFisher), penicillin/streptomycin (Gibco), and benzonase nuclease (EMD Millipore, Burlington, VT, USA). For livers, tissue was macerated through a 100-micron strainer and resuspended in a 35% percoll (Sigma) solution in PBS supplemented with 1500 units of heparin (Sigma) to enrich for leukocytes. For both liver and spleen suspensions, red blood cells were lysed with ACK lysis buffer (ThermoFisher) and resuspended in RPMI supplemented with 10% FBS. Cells were plated at 1 × 10^6^ to 3 × 10^6^ cells per well in a round bottom plate for further analysis.

Ex vivo stimulation for intracellular cytokine staining. For intracellular cytokine staining (ICS), single-cell suspensions were stimulated with RPMI 10% FBS media containing brefeldin A (BrfA) (5 µg/mL) (Biolegend, San Diego, CA, USA) only, brfA and a pool of overlapping 15-mer peptides derived from the CCHFV nucleoprotein covering amino acids 201–306 (1 µg/mL each peptide final concentration) [10] or brfA, phorbol 12-myristate-13-acetate (PMA) (81 nM), and ionomycin (5 µg/mL) (Biolegend). Cells were incubated at 37 °C with 5% CO_2_ for 4 h before proceeding with flow cytometry staining.

CD107a degranulation assay. Single-cell suspensions were resuspended in RMPI 10% FBS media supplemented with BrfA, monensin (Biolegend), and phycoerythrin (PE)-conjugated CD107a (clone 1D4B, 0.5 µg/mL final concentration, Biolegend). Cells received no stimulation or were stimulated with PMA and ionomycin or as above. Cells were incubated at 37 °C with 5% CO_2_ for 4 h before proceeding with flow cytometry staining.

Flow cytometry. Cells were washed and stained with Zombie Aqua viability dye (Biolegend) and Fc receptors blocked with TruStain FcX block (Biolegend). Cells were then stained with fluorophore-conjugated antibodies to CD3 (clone 145-2C11), CD4 (clone RM4.4), CD8 (clone 53-6.7), B220 (clone RA3-6B2), CD44 (clone IM7), CD62L (clone MEL-14), or CD69 (clone H1.2F3). For intracellular stains, cells were fixed and permeabilized with FOXP3 Transcription Factor staining buffer kit (ThermoFisher). Fixed and permeabilized cells were then stained with antibodies to Ki67 (clone 16A8), or for ICS: IFNγ (clone XMG1.2), TNFα (clone MP6-XT22), and interleukin-2 (IL-2, clone JES6-5H4). All antibodies were purchased from Biolegend or BD (San Jose, CA, USA). Cells were then washed and fixed with paraformaldehyde according to approved protocols for inactivation of CCHFV. Data was acquired using a FACSymphony instrument (BD) and data analyzed using FlowJo v10 software. Compensation was performed using UltraComp beads (eBioscience) and FlowJo v10 software. For all data, cells were gated to exclude debris, doublets, non-viable cells, and with a time gate. Cell populations were further identified as described in text.

Statistical Analyses. Indicated statistical tests were performed using Prism v8 (GraphPad).

## 3. Results

### 3.1. T-Cells in the Liver and Spleen Are Activated Following Cchfv Infection

**Figure 1:**
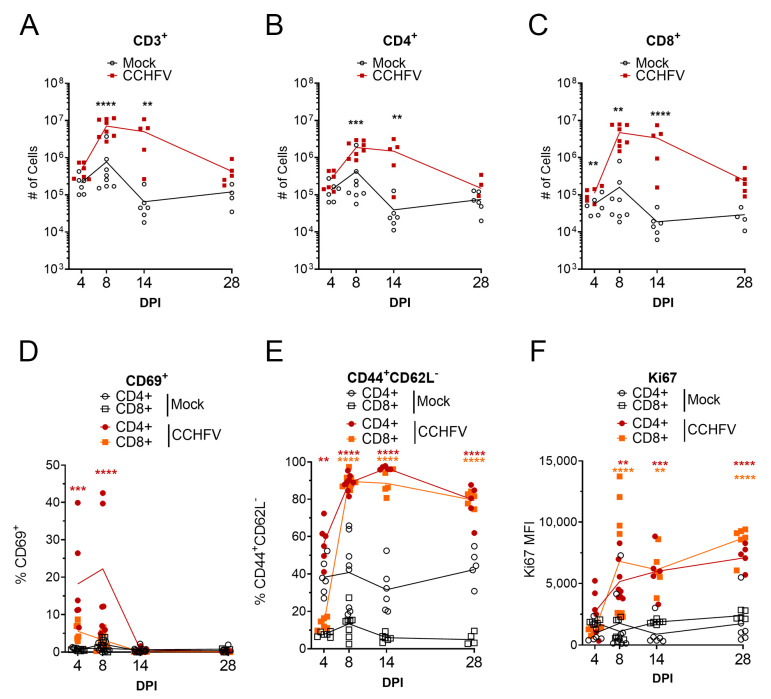
T-cells are robustly activated in the liver following Crimean-Congo hemorrhagic fever virus (CCHFV) infection. IFNAR^−/−^ mice were infected with CCHFV or mock-infected. At indicated timepoints, mice were euthanized and T-cell populations in the liver analyzed by flow cytometry. CD4^+^ or CD8^+^ T-cells were identified as CD3^+^B220^−^ and by exclusive expression of CD4 or CD8. (**A**–**C**) Data are presented as cell counts normalized to entire liver. (**D**,**E**) Data are presented at percentage of parental CD4^+^ or CD8^+^ T-cell populations. (**F**) The median-fluorescent intensity (MFI) of Ki67 is shown. (**A**–**F**) Data shown as individual data points with line connecting means. Statistical test comparing CCHFV-infected to mock-infected populations was performed using a two-way ANOVA with Sidak’s multiple comparison test. N = 5–8 per group. Statistical test comparing CD4 or CD8 populations from CCHFV-infected mice to respective populations in mock-infected mice was performed using a two-way ANOVA with Sidak’s multiple comparison test. N = 5–8 per group. ** *p* < 0.01, *** *p* < 0.001, **** *p* < 0.0001.

The primary targets of CCHFV replication in the IFNAR^−/−^ mouse model are the liver and spleen [10]. We hypothesized that infection of the liver and spleen would result in significant activation of the host adaptive immune response in these tissues. We further hypothesized that T-cell mediated immunity would be the primary host response that would control the primary infection. In the liver, compared to mock-infected mice, we observed significant increases in total number of CD3^+^ T-cells by day 8 PI in CCHFV-infected mice and increased numbers of cells were maintained until at least day 14 PI (Figure 1A). Similarly, compared to mock-infected mice, numbers of CD3^+^CD4^+^ and CD3^+^CD8^+^ T-cells in the livers of CCHFV-infected mice increased by day 8 PI and remained elevated until at least day 14 PI (Figure 1B,C). By day 28 PI, numbers of CD3^+^, CD3^+^CD4^+^, and CD3^+^CD8^+^ T-cells were similar (*p* > 0.05) to mock-infected mice (Figure 1A–C). In contrast to the livers, the spleens of the same mice showed no significant differences in numbers of total CD3^+^ or CD3^+^CD4^+^ T-cells (Supplementary Materials Figure S1A–C). However, the numbers of CD3^+^ CD8^+^ T-cells in the spleens of CCHFV-infected were significantly elevated at day 14 PI (Supplementary Materials Figure S1C).

We also evaluated the activation of T-cells in the liver and spleen by analyzing surface expression of the early activation marker CD69 [11]. In the liver, compared to mock-infected mice, the percentage of CD69^+^ CD4^+^ T-cells was significantly increased on days 4 and 8 PI (Figure 1D) indicating that CD4^+^ T-cells were rapidly activated following CCHFV infection. Interestingly, despite increases in the numbers of CD8^+^ T-cells in the livers of CCHFV-infected mice, we did not observe increased percentages of CD69^+^ CD8^+^ T-cells (Figure 1D). In the spleen, compared to mock-infected mice, percentages of CD69^+^ CD4^+^ and C69^+^ CD8^+^ T-cells were significantly increased at day 4 and percentages of CD69^+^ CD4^+^ remained significantly increased at day 8 PI (Supplementary Materials Figure S1D).

To further determine the activation status of the T-cells, we evaluated expression of CD44 and CD62-ligand (CD62L) and quantified the number of T-cells with the activated effector phenotype CD44^+^CD62L^−^ [12]. In the liver, compared to mock-infected mice, the percentage of CD4^+^ T-cells with the phenotype CD44^+^CD62L^−^ was significantly increased by day 4 PI (Figure 1E) and at day 8 and 14 PI, 90% or more of both CD4^+^ and CD8^+^ T-cells were CD44^+^CD62L^−^ (Figure 1E), suggesting substantial activation of CD4^+^ and CD8^+^ T-cells in this tissue. In the spleen, we observed a similar trend, with significant increases in the percentage of CD4^+^ and CD8^+^ T-cells of the phenotype CD44^+^CD62L^−^ at day 8 and 14 PI (Supplementary Materials Figure S1E). In both the liver and spleens of CCHFV-infected mice, the majority of CD4^+^ and CD8^+^ T-cells retained the CD44^+^CD62L^−^ activated effector phenotype at day 28 PI (Figure 1E and Supplementary Materials Figure S1E).

Lastly, we also evaluated the expression levels of Ki67 as a marker of cell proliferation [13]. By day 8 PI, in both the liver and spleen of CCHFV-infected mice, the median fluorescent intensity (MFI) of Ki67 in CD4^+^ and CD8^+^ T-cells was significantly increased compared to cells from mock-infected mice (Figure 1F). Ki67 MFI remained elevated as late as day 28 PI (Figure 1F). In the spleen, the Ki67 MFI was increased in both CD4^+^ and CD8^+^ T-cells at day 8 and 14 PI and remained elevated in CD8^+^ T-cells as late as day 28 PI (Supplementary Materials Figure S1F). These data suggested that both CD4^+^ and CD8^+^ T-cells were proliferating in the livers and spleen of CCHFV-infected mice. Cumulatively our data demonstrate that CCHFV infection results in robust and rapid activation of T-cells in both the liver and spleen.

### 3.2. T-Cells Produce T-Helper-1 (Th1)-Cytokines in Response CCHFV

A major function of T-cells during infection is production of cytokines to promote host defense against pathogens. We therefore utilized ICS to evaluate the ability of CD4^+^ and CD8^+^ T-cells from the livers of CCHFV-infected mice to produce the Th1-cytokines interferon gamma (IFNγ), tumor necrosis factor alpha (TNFα), and interleukin 2 (IL-2), which are associated with host defense against intracellular pathogens such as viruses [14]. We measured cytokine production in response to stimulation with a pool of peptides derived from the CCHFV nucleoprotein or PMA and ionomycin to identify their cytokine production potential.

**Figure 2:**
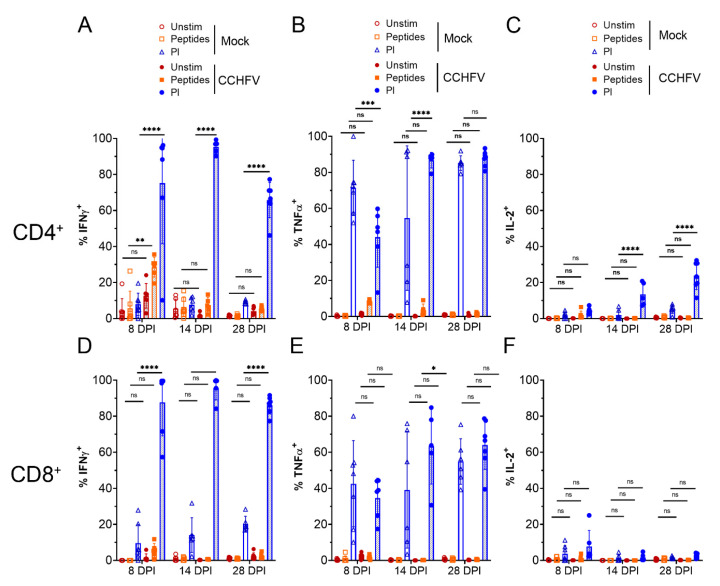
T-cells produce Th-1 cytokines in response to infection. IFNAR^−/−^ mice were infected with CCHFV or mock-infected. At indicated time points mice were euthanized, single cell suspensions generated from the liver and cytokine production by CD4^+^ T-cells (**A**–**C**) or CD8^+^ T-cells (**D**–**F**) measured by ICS and flow cytometry after ex vivo stimulation. CD4^+^ or CD8^+^ T-cells were identified as CD3+ and by exclusive expression of CD4 or CD8. Unstim = unstimulated. Peptides = peptide pool derived from the CCHFV NP. PI = PMA/Ionomycin. Data is presented as percentage of cytokine positive cells of parental CD4^+^ T-cells (**A**–**C**) or CD8^+^ T-cells (**D**–**F**). (**A**–**F**) N = 5–7 per group. Error bars indicate standard deviation. Statistical test comparing treatment groups between mock- and CCHFV-infected groups performed using two-way ANOVA with Tukey’s multiple comparison test. ns *p* > 0.05, * *p* < 0.05, ** *p* < 0.01, *** *p* < 0.001 **** *p* < 0.0001.

Compared to CD4^+^ T-cells from mock-infected mice, there was a significant increase in % IFNγ^+^ CD4^+^ T-cells at day 8 PI when stimulated with CCHFV peptides (Figure 2A), suggesting CD4^+^ T-cells were responding in a virus-specific manner. Compared to CD4^+^ T-cells from mock-infected mice, we otherwise did not observe significant increases in % TNFα^+^ or IL-2^+^ CD4^+^ T-cells in response to CCHFV peptides although there was a trend towards increased % TNFα^+^ at 8 DPI (Figure 2B). However, compared to cells from mock-infected mice, polyclonal stimulation of CD4^+^ T-cells with PMA/ionomycin resulted in significantly increased percentages of IFNγ^+^ CD4^+^ T-cells at 8, 14 DPI, and 28 DPI (Figure 2A). PMA/ionomycin stimulation resulted in the majority of CD4^+^ T-cells producing TNFα in both mock- and CCHFV-infected mice (Figure 2B). Lastly, we observed increased percentages of IL-2^+^ CD4^+^ T-cells of CCHFV-infected mice at 14 DPI and 28 DPI (Figure 2C) upon stimulation with PMA/ionomycin. Cumulatively, these data suggested that CD4^+^ T-cells from CCHFV-infected mice were rapidly primed to produce IFNγ by 8 DPI and IFNγ, TNFα, and IL-2 by 14 DPI and remained capable of producing these cytokines as late as day 28 PI.

We further evaluated cytokine production by CD8^+^ T-cells from the livers of infected mice. Compared to CD8^+^ T-cells from mock-infected mice, we observed no significant increases in % of IFNγ^+^, TNFα^+^, or IL-2^+^ CD8^+^ T-cells in response to CCHFV peptides (Figure 2D–F). However, we did observe significant increases in % IFNγ^+^ CD8^+^ T-cells at 8, 14, and 28 DPI upon polyclonal stimulation. Upon stimulation with PMA/ionomycin, at all timepoints evaluated, greater than 80% of CD8^+^ T-cells from CCHFV-infected mice were positive for IFNγ expression versus less than 20% of CD8^+^ T-cells from mock-infected mice (Figure 2D). We also found a significant increase in % TNFα^+^ CD8^+^ T-cells from CCHFV-infected mice at 14 DPI when stimulated with PMA/ionomycin (Figure 2E). Together, these data suggest that CD8^+^ T-cells in the livers of CCHFV-infected mice rapidly differentiate to produce IFNγ by day 8 PI.

### 3.3. T-Cells from CCHFV-Infected Mice Are Polyfunctional

For several infectious diseases, it has been found that T-cells capable of producing multiple cytokines simultaneously are functionally superior to T-cells capable of producing single cytokines [15,16,17,18]. We therefore determined the frequency of CD4^+^ and CD8^+^ T-cells from CCHFV-infected mice that produced one or more cytokines simultaneously following PMA/ionomycin stimulation. At day 8 PI, the CD4^+^ T-cell cytokine response to PMA/ionomycin stimulation was predominated by IFNγ single-producers (34%) and IFNγ/TNFα double-producers (36%) (Supplementary Materials Figure S2A). Thereafter, by day 14 PI, the CD4^+^ T-cell cytokine response was mostly IFNγ/TNFα double-producers (71%) (Supplementary Materials Figure S2B). However, by day 14 PI, 13% of CD4^+^ T-cells were IFNγ/TNFα/IL-2 triple-producers increasing to 22% by day 28 PI (Supplementary Materials Figure S2B,C). In addition, at day 28, we observed a significant fraction of TNFα single producers (24%) (Supplementary Materials Figure S2C). The CD8^+^ cytokine response to PMA/ionomycin stimulation was similar with mostly IFNγ single-producers (51%) and IFNγ/TNFα double-producers (30%) at day 8 PI (Supplementary Materials Figure S2D) followed by increasing percentages of IFNγ/TNFα double-producers (61%) at day 14 PI (Supplementary Materials Figure S2E). At day 28 PI, we still found a significant proportion of IFNγ single-producers (24%) although most were IFNγ/TNFα double-producers (50%) (Supplementary Materials Figure S2F). Together these data indicated that CCHFV-infection of mice induces a robust polyfunctional CD4^+^ and CD8^+^ T-cell response.

### 3.4. CD8^+^ T-Cells from CCHFV-Infected Mice Express High Levels of Perforin and Degranulate in Response to Stimulation

**Figure 3:**
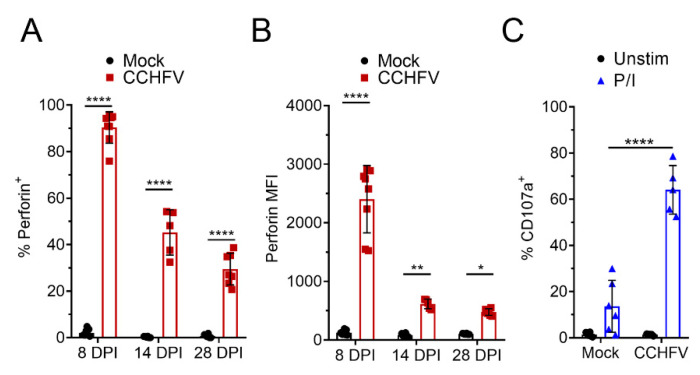
CD8^+^ T-cells from CCHFV-infected mice express perforin and degranulate. IFNAR^−/−^ mice were infected with CCHFV or mock-infected. At indicated time points, mice were euthanized, single cell suspensions generated from the liver, and analyzed by flow cytometry. (**A**–**C**) Liver CD8^+^ T-cells were identified as CD3^+^CD4^−^CD8^+^ and (**A**,**B**) intracellular perforin expression in CD8^+^ T-cells measured after 4 h ex vivo incubation in the presence of brfA. (**A**) Percent CD8^+^ T-cells positive for perforin expression and (**B**) the median fluorescent intensity (MFI) of all CD8^+^ T-cells is shown. (**A**,**B**) N = 5–8 per group. Error bars indicate standard deviation. Statistical comparison between CD8^+^ T-cells from CCHFV-infected mice (red) and mock-infected mice (open bar) was performed with a two-way ANOVA with Sidak’s multiple comparison test. (**C**) Degranulation of liver CD8^+^ T-cells at day 14 PI was monitored by CD107a staining. Percent CD107a^+^ CD8^+^ T-cells after indicated stimulation in the presence of brfA, monensin, and PE-conjugated CD107a is shown. Unstim = no stimulation. P/I = PMA and ionomycin. Error bars indicate standard deviation. N = 5 to 6 per group. Statistical comparison between CD8^+^ T-cells from CCHFV-infected mice and mock-infected mice was performed with a two-way ANOVA with Sidak’s multiple comparison test. * *p* < 0.05, ** *p* < 0.01, **** *p* < 0.0001.

A key function of CD8^+^ T-cells in control of viral infections is targeted killing of virally-infected cells and perforin- and granzyme-mediated killing is the primary mechanism by which cytotoxic T-cells kill target cells [19,20]. On day 8 PI, 90% of CD8^+^ T-cells in the liver of CCHFV-infected mice were positive for perforin expression (Figure 3A) and compared to CD8^+^ T-cells from mock-infected mice, had significantly increased expression of perforin (Figure 3B). At day 14 and 28 PI, we still observed significant increases in perforin^+^ CD8^+^ T-cells and perforin expression remained elevated (Figure 3A,B). Interestingly, at day 8 PI, compared to cells from mock-infected mice, we also observed a small but significant increase in perforin^+^ CD4^+^ T-cells from CCHFV-infected mice (Supplementary Materials Figure S3), suggesting that CD4^+^ T-cells may acquire some cytotoxic functions during CCHFV infection [21,22].

Upon recognition of a virally-infected cell, cytotoxic T-cells degranulate, releasing perforin and granzyme into the immunological synapse, resulting in death of the targeted cell [19]. To determine if perforin-expressing CD8^+^ T-cells could degranulate, at 14 days post-infection, we performed a CD107-degranulation assay [23,24]. Upon polyclonal stimulation with PMA/ionomycin, we observed a significant increase in CD107a^+^ CD8^+^ T-cells from CCHFV-infected mice (64%) compared to CD8^+^ T-cells from mock-infected mice (13%) (Figure 3C) suggesting most CD8^+^ T-cells in the livers of CCHFV-infected mice were primed to degranulate upon stimulation at this time point.

### 3.5. T-Cells Are Required for Survival of Acute CCHFV Infection

**Figure 4:**
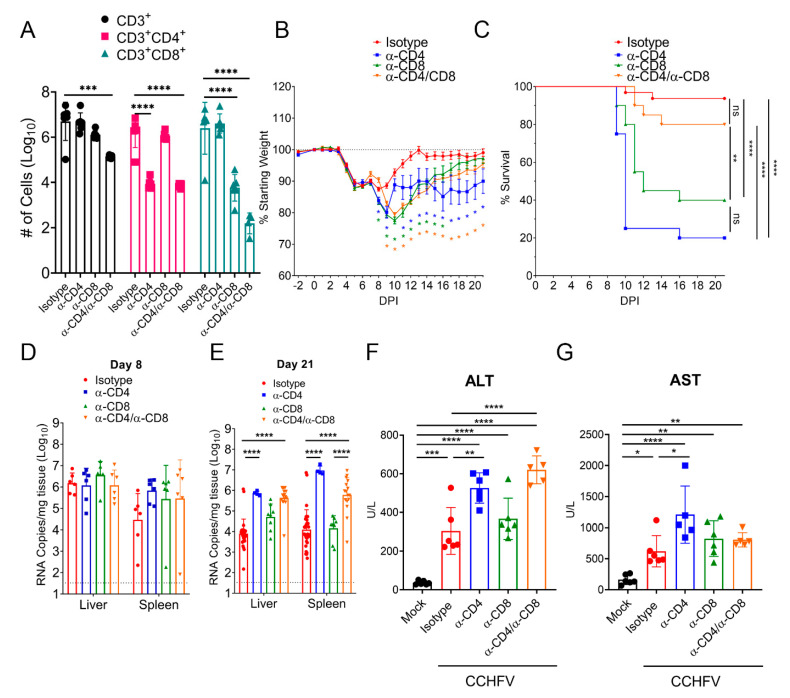
T-cells are required for survival following CCHFV infection. IFNAR^−/−^ mice were treated with antibodies to deplete CD4^+^, CD8^+^, or both CD4^+^ and CD8^+^ cells on day −2, +3, +10, and +17 relative to CCHFV challenge. (**A**) On day 8 PI, depletion efficacy was evaluated in a subset of mice by flow cytometry to enumerate number of CD3^+^, CD3^+^CD4^+^, or CD3^+^CD8^+^ T-cells in the liver. N = 6 per group. *p* values calculated with two-way ANOVA with Tukey-s multiple comparisons test. (**B**) Following CCHFV challenge, mice were weighed daily and monitored for survival (**C**). n = 20–32 mice per group. (**B**) Error bars indicate standard error of measurement. Statistical test comparing isotype-treated mice with depleted mice was performed using a two-way ANOVA with Dunnett’s multiple comparison test. (**C**) Indicated statistical comparison between groups performed using Log-rank test with Bonferroni’s correction for multiple comparisons. ** *p* < 0.01, **** *p* <0.0001. (**D**,**E**) At indicated time-points PI, mice were euthanized and viral RNA in the liver or spleen quantified by qRT-PCR. Dashed line indicates limit of detection. Statistical comparison to isotype-treated mice performed using two-way ANOVA with Sidak’s multiple comparison test. (**F**,**G**) At day 8 PI, blood levels of alanine aminotransferase (ALT) and aspartate aminotransferase (AST) were evaluated. Indicated statistical comparisons performed using an ordinary One-way ANOVA with Tukey’s multiple comparison test. ns *p* > 0.05, * *p* < 0.05, ** *p* < 0.01, *** *p* < 0.001, **** *p* < 0.0001.

Cumulatively our data indicated that CCHFV infection in mice resulted in activation, proliferation, and production of antiviral cytokines by T-cells in the liver. To determine the contribution of this response to survival in CCHFV-infected mice, we depleted CCHFV-infected mice of either CD4^+^ or CD8^+^ T-cells or both. Our depletion regimen resulted in significant depletion of targeted CD4^+^, CD8^+^, or both T-cell populations in the liver at day 8 PI and depletion of one T-cell subset had no significant impact on numbers of the non-targeted subset (Figure 4A). Compared to isotype-control treated mice, mice depleted of CD4^+^, CD8^+^, or both CD4^+^ and CD8^+^ T-cells had significantly exacerbated clinical disease as evidenced by significantly increased and prolonged weight loss, with mice depleted of CD4^+^ or both CD4^+^ and CD8^+^ T-cells still exhibiting weight loss at study end on day 21 PI (Figure 4B). Furthermore, mice depleted of CD4^+^ or CD8^+^ T-cells had significantly increased mortality with only 20% of CD4^+^ T-cell depleted (mean-time-to-death (MTD) = day 10 PI) and 40% of CD8^+^ T-cell depleted mice (MTD = day 12 PI) surviving the acute infection (Figure 4C). Interestingly, although combined CD4 and CD8 depletion resulted in exacerbated and prolonged weight loss compared to isotype-control treated mice (Figure 4B), survival in these mice was not significantly worsened compared to isotype-treated mice (Figure 4C). Survival of combined CD4 and CD8 depleted mice was significantly improved compared to mice depleted of either CD4^+^ or CD8^+^ T-cells alone (Figure 4C), indicating absent T-cell responses results in improved outcome compared to partial T-cell responses. However, compared to isotype-treated mice, mice depleted of both CD4^+^ and CD8^+^ T-cells had significantly exacerbated and prolonged clinical disease (Figure 4B), demonstrating T-cells contribute to resolution of acute CCHFV infection.

We next evaluated whether depletion of T-cells resulted in uncontrolled viral replication. We measured viral RNA loads in the liver and spleen at day 8 PI (Figure 4D) and in surviving mice at study end on day 21 PI (Figure 4E). Interestingly, we found that viral RNA loads in the liver and spleens of T-cell depleted mice were not significantly different from isotype-control treated mice at day 8 PI (*p* > 0.05) (Figure 4D). However, consistent with the prolonged weight loss observed in surviving CD4^+^ T-cell depleted mice (Figure 4B), at day 21 PI, mice depleted of CD4^+^ or both CD4^+^ and CD8^+^ T-cells had significantly increased viral RNA loads in the liver and spleen compared to isotype treated mice (Figure 4E). These data suggest that an absence of CD4^+^ T-cells results in an eventual failure to control the virus in these tissues. Lastly, at day 8 PI, we evaluated blood levels of alanine aminotransferase (ALT) and aspartate aminotransferase (AST) as markers of liver damage in these mice. CCHFV-infected mice had significantly increased levels of AST and ALT compared to mock-infected mice (Figure 4F,G). Consistent with the more severe disease in CD4-depleted mice, these mice had significantly increased levels of AST and ALT compared to isotype-treated CCHFV-infected mice (Figure 4F,G). In contrast, CD8-depleted mice did not have significantly increased AST or ALT levels compared to isotype-treated CCHFV-infected mice (Figure 4F,G). Interestingly, mice depleted of both CD4^+^ and CD8^+^ T-cells had significantly increased levels of ALT but not AST compared to isotype-treated CCHFV-infected mice (Figure 4F,G).

### 3.6. CD4^+^ T-Cell Depletion Impairs Early Antibody Responses but Not Early CD8^+^ T-Cell Responses

**Figure 5:**
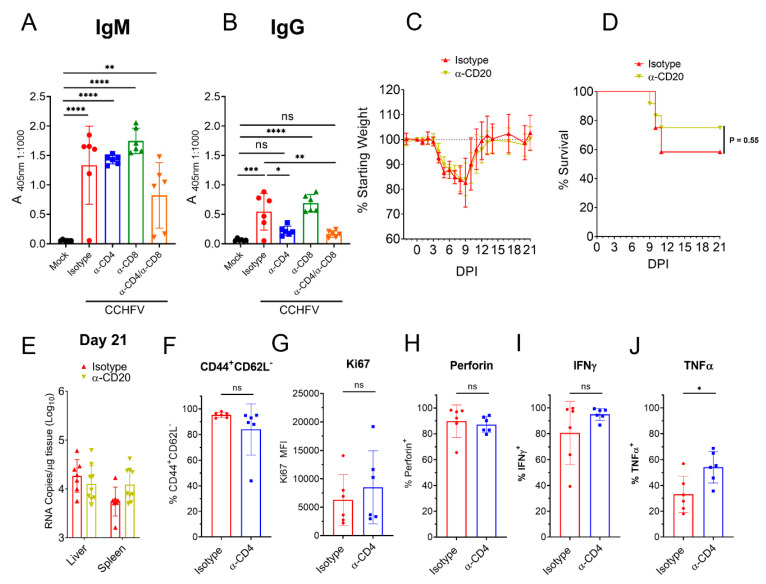
Depletion of CD4^+^ T-cells impairs early antibody but not early CD8^+^ T-cell responses. IFNAR^−/−^ mice were infected with CCHFV and on day +8 PI CCHFV-specific IgM (**A**) or IgG (**B**) measured by whole-virion ELISA. N = 5–6 per group. Indicated statistical comparison performed with one-way ANOVA with Tukey’s multiple comparisons test. (**C**–**E**) CCHFV-infected IFNAR^−/−^ mice were treated with α-CD20 or isotype control and weighed daily (**C**) and monitored for survival (**D**). (**C**,**D**) N = 12 per group. (**D**) *p* value calculated with Log-rank test. (**E**) At day 21 PI, viral loads in liver and spleen quantified by qRT-PCR. N = 6 per group. (**F**–**J**) At day 8 PI, CD8 T-cells in the liver of IFNAR^−/−^ mice treated with isotype or antibody to deplete CD4 T-cells were evaluated for their surface expression of CD44, CD62L (**F**) and expression of Ki67 (**G**). (**H**) CD8 T-cells were analyzed for their expression of perforin after 4 h incubation in presence of brfA. (**I**,**J**) CD8 T-cells were analyzed for their expression of IFNγ and TNFα after stimulation with PMA/ionomycin. (**F**–**J**) N = 6 per group. Statistical test performed with unpaired *t*-test. ns *p* > 0.05, * *p* < 0.05, ** *p* < 0.01, *** *p* < 0.001, **** *p* < 0.0001.

CD4^+^ T-cells can support B-cell and CD8^+^ T-cell responses through direct and indirect mechanisms [25,26] and we hypothesized that poor outcome in mice depleted of CD4^+^ T-cells may be due to impaired B- cell or CD8^+^ T-cell responses. Although depletion of CD4^+^ T-cells did not impair early CCHFV-specific IgM at day 8 PI, CD4^+^ T-cell depleted mice had diminished IgG responses (Figure 5A,B) suggesting the early IgG response to CCHFV is T-cell-dependent. To determine if B-cells were needed for survival, we depleted CCHFV-infected mice of B-cells using α-CD20 depletion. CCHFV-infected mice treated with α-CD20 had similar weight loss, survival and viral loads in the liver and spleen at day 21 PI as mice treated with an isotype-control, suggesting B-cells are not necessary for survival following acute infection (Figure 5C–E). However, despite our depletion regimen significantly depleting B-cells in the spleens of CCHFV-infected mice at day 5 PI (Supplementary Materials Figure S4A), α-CD20 treatment diminished but did not completely abolish a CCHFV-specific antibody response at day 21 PI (Supplementary Materials Figure S4B). Thus, we cannot rule out that even these diminished antibody responses may contribute to survival of acute infection.

Although depletion of CD4^+^ T-cells did not impact numbers of CD8^+^ T-cells to the liver, it is possible that activation or effector functions of CD8^+^ T-cells was impaired in CD4^+^ T-cell depleted mice. At day 8 PI, we analyzed the activation status and ability to produce IFNγ and TNFα by liver CD8^+^ T-cells from CCHFV-infected mice depleted of CD4^+^ T-cells. We found compared to mice with intact CD4^+^ T-cell responses, mice depleted of CD4^+^ T-cells had similar percentages of liver CD8^+^ T-cells with the activated effector phenotype (Figure 5F) and similar expression of Ki67 (Figure 5G). Furthermore, mice depleted of CD4^+^ T-cells had similar percentages of perforin^+^ and IFNγ^+^ CD8^+^ T-cells in the liver (Figure 5H,I) and a small but significant increased percentage of TNFα^+^ CD8^+^ T-cells (Figure 5J). Together, these data indicated that depletion of CD4^+^ T-cells impaired early CCHFV-specific IgG but did not impair the acute CD8^+^ T-cell response to the infection in the liver by the criteria measured.

### 3.7. IFNγ Is Required for Survival Following CCHFV Infection

**Figure 6:**
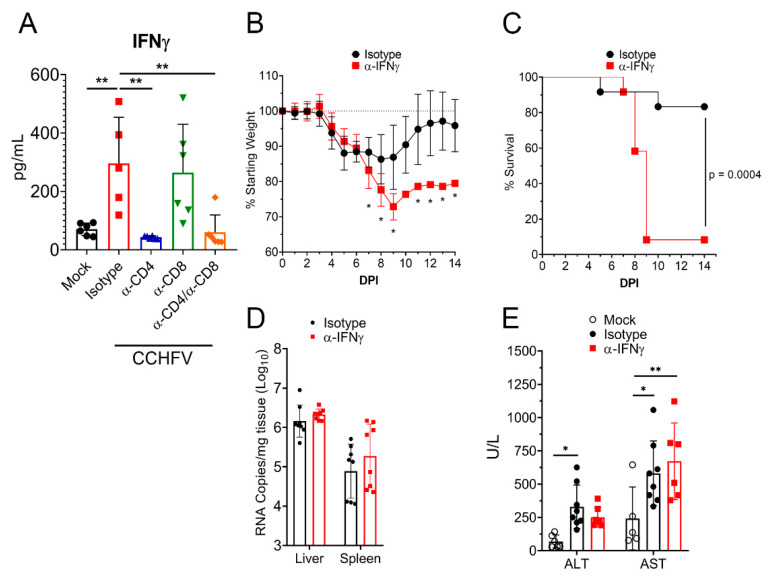
IFNγ is required for survival following CCHFV infection. (**A**) IFNAR^−/−^ mice were infected with CCHFV or mock-infected. Mice were treated as indicated with isotype-control antibody, antibody to deplete CD4 or CD8 T-cells or both. On day 8 PI, plasma IFNγ levels in mice were measured by Bio-plex assay. Statistical comparison performed with a one-way ANOVA and Tukey’s multiple comparison test. (**B**–**D**) IFNAR^−/−^ mice were infected with CCHFV and treated with isotype-control antibody or α-IFNγ antibody to neutralize IFNγ. Mice were weighed daily (**B**) and euthanized according to criteria outlined in methods (**C**). (**B**) N = 12 per group. Statistical comparison between isotype-control treated and α-IFNγ-treated mice performed with a two-way ANOVA with Sidak’s multiple comparison test. * *p* < 0.05. (**C**) N = 12 per group. Statistical test between survival curves performed using Log-rank test. (**D**) At day 8 PI, viral RNA loads in the liver or spleen were quantified by qRT-PCR. Statistical test comparing isotype-control treated and α-IFNγ-treated mice performed using two-way ANOVA with Sidak’s multiple comparison test. *p* > 0.05. (**E**) At day 8 PI, in mock-infected or CCHFV-infected mice treated with isotype control or IFNγ neutralizing antibody, blood levels of alanine aminotransferase (ALT) and aspartate aminotransferase (AST) were evaluated. Indicated statistical comparisons performed using an ordinary Two-way ANOVA with Tukey’s multiple comparison test. * *p* < 0.05, ** *p* < 0.01.

Our data demonstrated that T-cells were required for survival from acute CCHFV infection. IFNγ is a key antiviral cytokine produced by activated T-cells with numerous antiviral functions [27,28] and ICS analysis of T-cells following CCHFV infection demonstrated that CD4^+^ and CD8^+^ T-cells from the livers of CCHFV-infected mice rapidly differentiate to produce IFNγ. We therefore hypothesized that depletion of T-cells may impair the IFNγ response to the CCHFV infection. Indeed, when we analyzed plasma IFNγ levels in CCHFV-infected mice at day 8 PI, levels in CCHFV-infected CD4 or CD4 and CD8-depleted mice were similar to mock-infected mice (*p* > 0.05), suggesting depletion of CD4^+^ T-cells completely abrogated the plasma IFNγ response to CCHFV (Figure 6A).

To determine if IFNγ was required for survival of acute CCHFV infection, we treated mice with an antibody to neutralize IFNγ activity in vivo [29] or isotype-control and then infected the mice with CCHFV. Mice treated with the IFNγ-neutralizing antibody had significantly exacerbated clinical disease as evidenced by significantly increased weight loss (Figure 6B). This increased weight loss correlated with worsened survival, as 11 of 12 mice treated with the IFNγ-neutralizing antibody succumbed to the infection versus only 2 of 12 mice treated with the isotype-control (*p* = 0.0004) (Figure 6C). Similar to our T-cell depletion data, viral loads at day 8 PI were not significantly different (*p* > 0.05) between isotype-control and IFNγ neutralizing antibody treated mice (Figure 6D). However, in contrast to mice depleted of CD4^+^ T-cells, mice treated with the IFNγ neutralizing antibody did not exhibit significantly increased liver enzymes compared to isotype-treated mice (Figure 6E), suggesting neutralization of IFNγ did not significantly influence liver damage. These data were again surprising as day 8 PI was shortly before death in mice with neutralized IFNγ (MTD = day 9 PI), demonstrating that even shortly before death, absence of IFNγ did not significantly impact control of acute CCHFV replication.

## 4. Discussion

Our understanding of the host adaptive immune responses necessary to control CCHFV infection is limited. Several studies in humans have identified that low-to-absent early antibody responses to the virus correlate with poor outcome [3,5,30]. However, it remains unclear if the failure to mount an antibody response to the infection results in uncontrolled viral infection and death or if the failure to mount an antibody response is merely a correlate of an ineffective immune response. Even among survivors, antibody responses may be poorly neutralizing [5] and in mice, a vaccine that elicited neutralizing antibody responses failed to confer protection against a lethal challenge [30]. In another study, both humoral and cellular mediated immunity were required for vaccine-mediated protection [31]. These findings suggest that antibody responses on their own may be insufficient to control CCHFV infection. If or how T-cells contribute to control of CCHFV is similarly poorly understood. Human survivors generate memory T-cell responses [32] and fatal cases had elevated levels of circulating CD8^+^ T-cells [33]. In one study, fatal human cases of CCHF had elevated levels of IL-10 while survivors had elevated levels of IL-12, suggesting weak Th1-type immune responses may contribute to fatal disease [7]. In humanized mice infected with CCHFV, CD4^+^, and CD8^+^ T-cells had increased expression of activation markers and CD8^+^ T-cells in terminal but not surviving mice had increased levels of perforin [34]. Nevertheless, it remains unclear the contribution of T-cells in control and resolution of a primary CCHFV infection in naïve mice.

Our data identify a role for both CD4^+^ and CD8^+^ T-cells in survival of acute CCHFV infection and demonstrate that both CD4^+^ and CD8^+^ T-cells are rapidly primed to engage in antiviral functions. CD4^+^ T-cells can promote effective B-cell and CD8^+^ T-cell responses through licensing of APCs [25], recruitment of CD8^+^ T-cells to the sites of infection [35], and direct interaction with these cell types [36]. However, analysis of the CD8^+^ T-cell response during acute infection demonstrated that depletion of CD4^+^ T-cells did not impair recruitment, activation, or cytokine production by CD8^+^ T-cells in the liver. CD4^+^ T-cells can also have direct antiviral roles through production of IFNγ [37,38]. Although, CD4^+^ T-cells were necessary for the systemic IFNγ response and IFNγ signaling was required for survival, neither depletion of CD4^+^ T-cells nor blockade of IFNγ resulted in consistently increased viral loads in the liver or spleen suggesting activities other than direct restriction of viral replication.

We found that mice depleted of CD4^+^ T-cells had diminished early antibody responses to CCHFV, suggesting that CD4^+^ T-cells may contribute to survival through support of early antibody responses to the infection. Depletion of B-cells did not impact survival or later viral loads, suggesting antibody may be dispensable for control of the acute infection. However, our depletion regimen was unable to completely block development of CCHFV-specific antibody responses. Thus, it is possible even these diminished responses contribute to control of the infection. We also demonstrated a significant role of CD8^+^ T-cells in survival of CCHFV infection. Localized production of IFNγ by CD8^+^ T-cells within infected tissues may contribute to survival following infection and it is also possible that CD8^+^ T-cells contribute to control of CCHFV through targeted killing of infected cells. CD8^+^ T-cells from CCHFV-infected mice constitutively expressed perforin and were capable of degranulating suggesting they were primed to kill target cells. The precise effector functions of CD4^+^ and CD8^+^ T-cells required for survival of acute CCHFV infection will require further study.

We were surprised that although depletion of T-cells or blockade of IFNγ resulted in significantly worsened disease, these treatments did not consistently impact viral loads in the liver or spleen at day 8 PI. These data suggest that fatal disease outcome in these mice may not result from uncontrolled viral replication and instead may be due to dysregulated immune responses. CD8^+^ T-cells have been shown to limit CD4^+^ T-cell mediated immunopathology following pulmonary infection with respiratory syncytial virus [39] and CD4^+^ T-regulatory cells have been shown to restrain activation, proliferation, and effector functions of numerous host immune cells [40]. IFNγ is a multifunctional cytokine exhibiting both direct antiviral and immunomodulatory effects [28,41,42]. Early IFNγ responses by NK cells may enhance APC function of dendritic cells, promote killing of intracellular pathogens by macrophages, and polarize CD4^+^ T-cells to become T helper 1 cells whose subsequent IFNγ responses engage in a positive feedback loop to promote effective control of the pathogen [43]. Alternatively, IFNγ can also suppress B- and CD8^+^ T-cell responses [41,42] and absent IFNγ signaling could lead to T-cell mediated pathology. IFNγ-deficient mice infected with lymphocytic choriomeningitis virus developed a severe CD8^+^ T-cell mediated immunopathology [44] and responding CD8^+^ T-cells unable to produce IFNγ led to severe pulmonary immunopathology in influenza-infected mice [45]. We also cannot exclude the possibility that uncontrolled viral replication may have occurred at tissues other than the liver and spleen or may have occurred at later timepoints prior to mice succumbing to CCHFV. Indeed, CD4^+^ T-cell deficient mice that survived the acute infection had significantly elevated viral loads at day 21 PI, demonstrating that these mice eventually fail to control viral replication. Lastly, CCHFV-infected mice depleted of CD4^+^ T-cells had significantly elevated liver enzymes compared to infected mice with intact responses, suggesting increased liver damage in mice lacking CD4^+^ T-cells. In contrast, although mice depleted of CD8^+^ T-cells succumbed to the infection, these mice did not exhibit increased liver enzymes compared to isotype-treated mice at the timepoint evaluated. Similarly, mice with neutralized IFNγ did not exhibit increased viral loads in the liver or spleen nor increased liver enzymes compared to isotype-treated mice just one day prior to succumbing to the infection. Cumulatively, further studies are needed to precisely define why mice with absent T-cells or IFNγ signaling succumb to the infection.

An important limitation of our study is the lack of type I IFN signaling in the mice. CCHFV-infection of mice with intact type I IFN signaling results in no clinical disease and severely restricted viral replication [46], making them unsuitable for studying the host response to severe CCHFV infections. Lack of type I IFN can significantly impact T-cell responses either through direct effects on T-cells themselves or indirectly through effects on antigen presenting cells necessary for activation of T-cells [47]. Type I interferons can provide the “third” activating signal for CD8 T-cells to undergo expansion and differentiation [48]. Type I IFNs can also suppress T-cells through “out-of-sequence” signaling by the T-cell receptor and type I IFN receptor [47], a mechanism which cannot occur in our mouse model. Further, lack of type I IFN signaling can negatively impact the function of antigen presentation cells such as dendritic cells through diminished expression of MHC-I, MHC-II, and costimulatory molecules [47,49]. In the context of vaccine-development, several studies have identified altered T-cell responses in vaccinated wild-type versus IFNAR^−/−^ mice [50]. In addition, to altered T-cell immunity, IFNAR^−/−^ mice can also exhibit altered humoral immunity to vaccines [50]. However, in at least one study evaluating a CCHFV-vaccine, although there were slight differences in vaccine responses, no difference was found in disease outcome between vaccinated IFNAR^−/−^ or transiently IFN suppressed mice [51], demonstrating that type I IFN signaling is dispensable for development of protective vaccine-mediated responses against CCHFV. Given the key interaction between type I IFN signaling and adaptive immunity, our findings presented here must be carefully considered in the context of type I IFN-competent hosts, e.g., humans. Nevertheless, our data demonstrate that even in the absence of type I IFN signaling, CD4^+^ and CD8^+^ T-cells are robustly activated and can prevent mortality following acute CCHFV infection.

In conclusion, we have identified that T-cells are robustly activated and required for survival following CCHFV-infection in mice. Furthermore, we have identified that IFNγ is a key cytokine necessary for survival in CCHFV-infected mice. Cumulatively, we have identified host responses necessary for survival of acute CCHFV infection and have expanded our understanding of how the host responds to the infection. Importantly, studies to precisely define the T-cell effector functions required for survival in CCHFV-infected mice, to define how IFNγ promotes survival in these mice and how these responses develop in hosts with intact type I IFN are needed. Our findings presented here will guide studies in the recently developed immunocompetent mouse model for CCHF [52] and in the cynomolgus macaque model [53] that recapitulate many aspects of human disease in hosts with intact innate immunity. Together, these data will inform therapeutic strategies to promote protective immune responses, limit pathogenic responses, and determine how viral–host interactions lead to the significant morbidity and mortality in CCHFV-infected humans.

## Data Availability

Data presented in this study is available upon request.

## Supplementary Materials

The following are available online at https://www.mdpi.com/2076-2607/9/2/279/s1, Figure S1: T-cells are robustly activated in the spleen, Figure S2: T-cells from the liver of CCHFV-infected mice produce multiple cytokines, Figure S3: CD4 T-cells express perforin in CCHFV-infected mice, Figure S4: Efficacy of B-cell depletion in CCHFV-infected mice.
