# Supplementary material for: T-Cells and Interferon Gamma Are Necessary for Survival Following Crimean-Congo Hemorrhagic Fever Virus Infection in Mice
Source: Microorganisms. 2021 Jan 29;9(2):279. doi: 10.3390/microorganisms9020279 (PMC7912317; doi:10.3390/microorganisms9020279)
Supplement: Supplementary file 1 [file microorganisms-09-00279-s001.pdf]

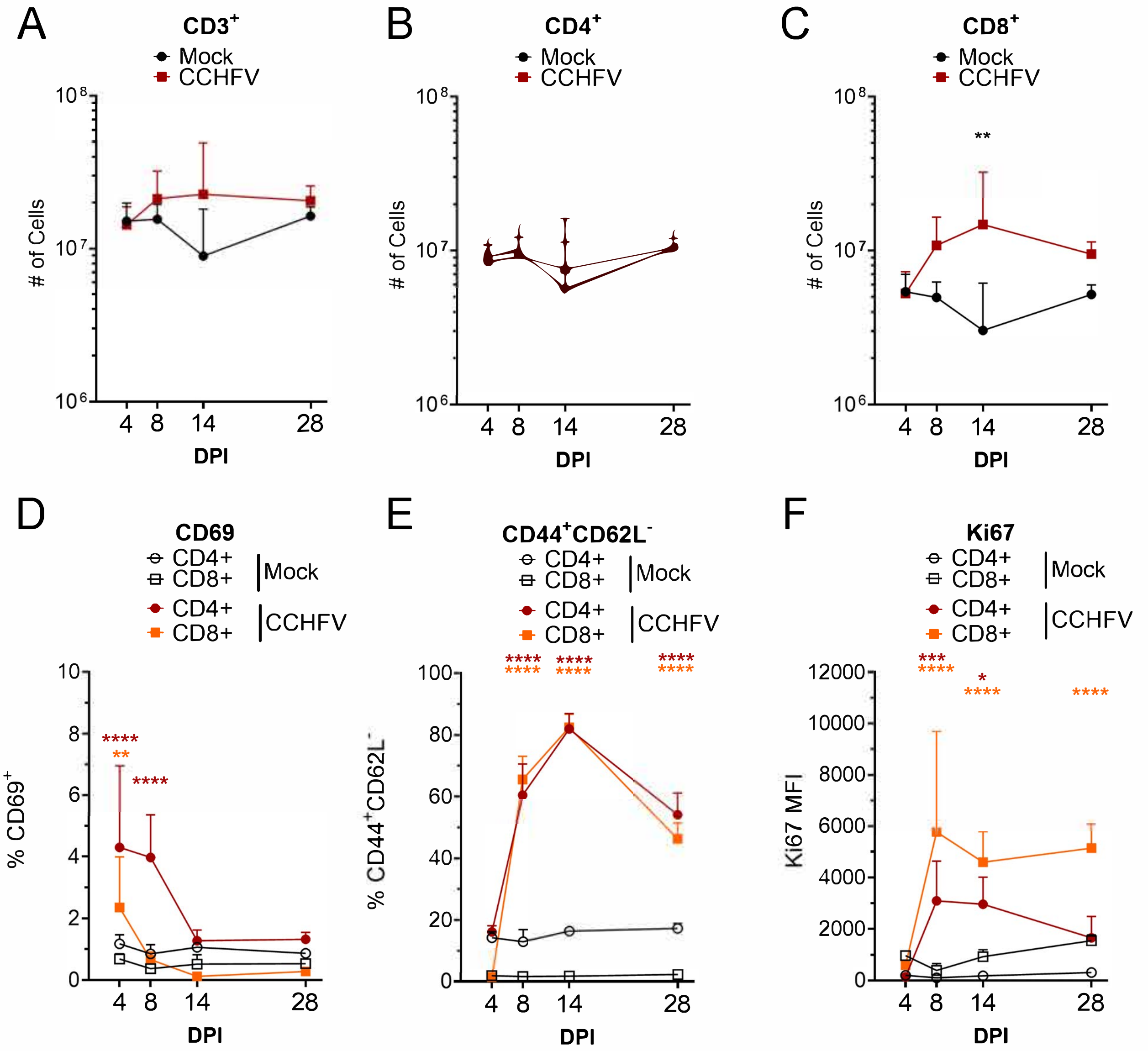

Supplemental Figure S1: T-cells are robustly activated in the spleen following CCHFV infection. IFNAR-/- mice were infected with CCHFV or mock-infected. At indicated timepoints, mice were euthanized and T-cell populations in the spleen analyzed by flow cytometry. CD4+ or CD8+ T-cells were identified as CD3+B220- and by exclusive expression of CD4 or CD8. (A – C) Data is presented as cell counts normalized to entire spleen. (D & E) Data is presented at percentage of parental CD4+ or CD8+ T-cell populations. (F) The median-fluorescent intensity (MFI) of Ki67 is shown. (A – C) Statistical test comparing CCHFV-infected to mock-infected populations was performed using a two-way ANOVA with Sidak's multiple comparison test. (D – F) Statistical test comparing CD4 or CD8 populations from CCHFV-infected mice to respective populations in mock-infected mice was performed using a two-way ANOVA with Sidak's multiple comparison test. *p<0.05, ** p <0.01, *** p <0.001, **** p <0.0001

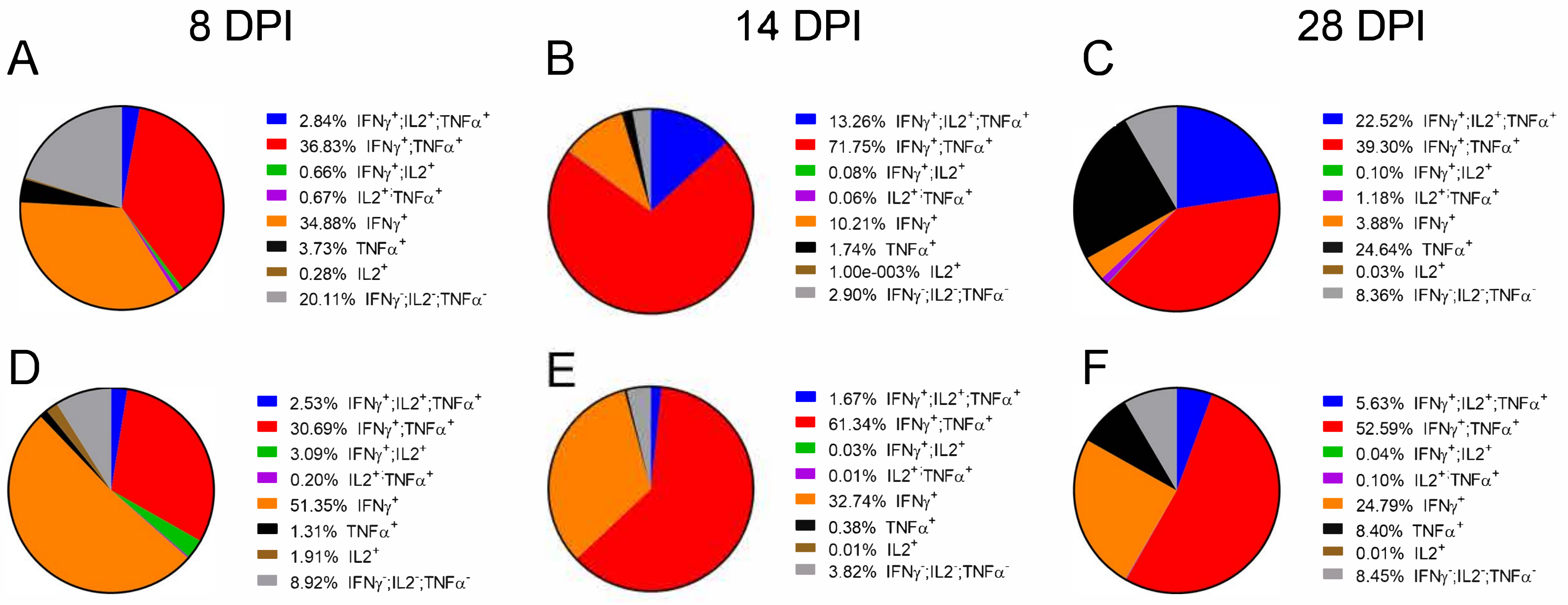

Supplemental Figure S2: T-cells from the liver of CCHFV-infected mice produce multiple cytokines. IFNAR-/- mice were infected with CCHFV and at day 8 (A & D), 14 (B & E) and 28 (C & F) post-infection, cytokine expression by CD4 (A - C) and CD8 T-cells (D - F) evaluated by ICS and flow cytometry after ex vivo stimulation with PMA/Ionomycin. Boolean gates were used to determine percentage of parental CD4 or CD8 population expressing one or more cytokines.

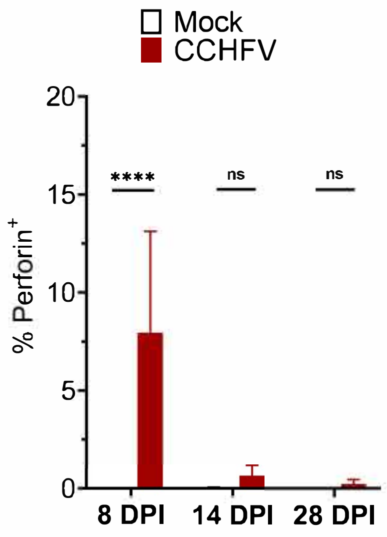

Supplemental Figure S3: CD4 T-cells express perforin in CCHFV-infected mice. IFNAR-/- mice were infected with CCHFV or mock-infected. At indicated time points mice were euthanized, single cell suspensions generated from the liver and analyzed by flow cytometry. Liver CD4+ T-cells were identified as CD3+CD4+CD8- and intracellular perforin expression in CD4+ T-cells measured after 4 hours ex vivo incubation in the presence of brfA. Statistical tests performed using two-way ANOVA with Sidak's multiple comparison test. **** $p < 0.0001$. ns p

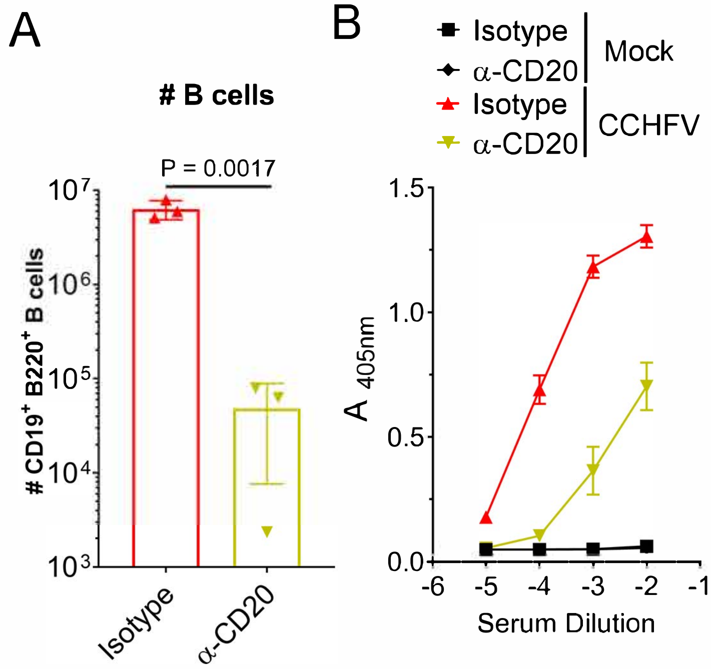

Supplemental Figure S4: Efficacy of B-cell depletion in CCHFV infected mice. Groups of IFNAR-/- mice were infected with CCHFV and treated with α-CD20 or isotype control. (A) On day +5 number of B-cells in the spleen were enumerated. P value calculated with unpaired t-test. (B) CCHFV-specific Ig was quantified by whole-virion ELISA on serum colected at day +21 PI.
